# The Influence of Peer Reviewer Expertise on the Evaluation of Research Funding Applications

**DOI:** 10.1371/journal.pone.0165147

**Published:** 2016-10-21

**Authors:** Stephen A. Gallo, Joanne H. Sullivan, Scott R. Glisson

**Affiliations:** Scientific Peer Advisory and Review Services Division, American Institute of Biological Sciences, Reston, Virginia, United States of America; Technion Israel Institute of Technology, ISRAEL

## Abstract

Although the scientific peer review process is crucial to distributing research investments, little has been reported about the decision-making processes used by reviewers. One key attribute likely to be important for decision-making is reviewer expertise. Recent data from an experimental blinded review utilizing a direct measure of expertise has found that closer intellectual distances between applicant and reviewer lead to harsher evaluations, possibly suggesting that information is differentially sampled across subject-matter expertise levels and across information type (e.g. strengths or weaknesses). However, social and professional networks have been suggested to play a role in reviewer scoring. In an effort to test whether this result can be replicated in a real-world unblinded study utilizing self-assessed reviewer expertise, we conducted a retrospective multi-level regression analysis of 1,450 individual unblinded evaluations of 725 biomedical research funding applications by 1,044 reviewers. Despite the large variability in the scoring data, the results are largely confirmatory of work from blinded reviews, by which a linear relationship between reviewer expertise and their evaluations was observed—reviewers with higher levels of self-assessed expertise tended to be harsher in their evaluations. However, we also found that reviewer and applicant seniority could influence this relationship, suggesting social networks could have subtle influences on reviewer scoring. Overall, these results highlight the need to explore how reviewers utilize their expertise to gather and weight information from the application in making their evaluations.

## Introduction

Despite the nearly ubiquitous use of scientific peer review to help guide the highly competitive allocation of billions of dollars in research funding and long-standing concerns about the reliability and validity of review results, peer review remains under-studied, particularly in regard to the types of decision-making processes reviewers use in the evaluation of applications [1–8]. Historically, the relative agreement between reviewers assessing the same application has been reported to be quite low [8–10]. Cole et al. (1981) has suggested that reviewer disagreement can, in part, be attributed to the process of translating opinion to score as well as epistemological differences in the quality of science (among individuals and among fields), while many studies have suggested a variety of individual biases may potentially be at play [8,11]. Whatever the source, it is clear that there is a great deal of subjectivity in the evaluation of research applications and understanding the sources of (and relative contributions to) reviewer disagreement will be crucial to improve the peer review process.

Individual differences in decision-making, as with bias, can be influenced by investigator and reviewer characteristics, as well as the content of the scientific work and limitations in assessing the true quality of research [11]. One important, but often neglected, reviewer characteristic is subject matter expertise. While there is a substantial literature on the use of expertise in decision-making, relatively little has been published on how a reviewer’s scientific expertise contributes to their decision-making processes in peer review [12–14]. Currently, most funding agencies utilize evaluators with close subject matter expertise relative to the applications that are being reviewed, as it is believed that recruitment of appropriate expertise is vital to the legitimacy of peer review [15]. Indeed, it has been noted that deference to expertise is an important aspect of peer review [16].

Two recent studies have explored the relationship between the proximity of applicant and reviewer and scoring behavior, and have found contrasting results. The first study, a blinded, randomized experimental peer review of biomedical research funding applications (Boudreau et al., 2016), examined individual judgments relative to the intellectual distance between the research applications and reviewers (through comparisons of associated medical subject heading [MeSH] keywords) and found that reviewers with a shorter intellectual distance from the application tended to provide harsher evaluations, even on work that is highly innovative [17]. Based on the observed linear nature and the direction of the relationship, the authors discount popular theories where reviewers are either motivated to promote close research based on similar schools of thought or cronyism (opposite directionality) or motivated by strategic incentives to penalize “close” research competing for resources (non-linearity) [18–19]. Also, classical theories of decision making under uncertainty are discounted as they predict opposite directionality [20]. Therefore, the authors explain these occurrences through a proposed bounded rationality decision-making model [21]. In this model, reviewers’ rational decisions are limited by constraints of computational resources and in the availability of information; in this case, information is limited to what can be gathered by the reviewer from the research application and his/her focused knowledge in their area(s) of expertise [17]. The authors suggest that reviewers with higher expertise sample more information from the application, detecting more weaknesses than non-experts, which may lead to harsher evaluations. While other studies have implicated a tendency of reviewers to focus and/or agree more on weaknesses than strengths, the amplification of this tendency based on expertise has important implications on the promotion of innovative research and on reviewer recruitment [9,10].

These results are contrasted by the results from a retrospective study of data from the unblinded NIH grant review process by Li (2015), which suggest that a higher degree of relatedness between the applicant and review committee members (by way of reviewers citing the applicant’s work in their publications) yields a greater probability of being funded [22]. This result is directly opposed to the above findings, however, there are some important differences. The Boudreau study was an experimental study blinded to applicant identity and investigated individual reviewer scoring while the Li analysis was an analysis of historical data from unblinded reviews and examined overall scoring by study section panel (which included panel dynamics and discussion effects). An important additional difference is in the measure of proximity between reviewer and applicant; the first study using a direct measure of topic area similarity (intellectual distance) between reviewer and applicant and the second study utilizing a measure of citation behavior of reviewers relative to the applicant (relatedness). Li contends that citation relatedness allows reviewers to sample important information about the quality of the application through knowledge of the applicant’s body of work. In this sense, citation relatedness is not necessarily the same as intellectual distance, and may be influenced by social network strength and status.

While both status and social networking have been examined for their potential role in research funding and peer review [15,23–28], in the work of Boudreau the review was blinded to applicant identity, precluding any social and professional networking effects. Therefore, it is unclear how subject matter expertise affects reviewer evaluations in cases where social networking effects may apply. Bounded rationality may still be a consistent explanation for the role of expertise in this case, but if social networking effects dominate, the relationship between expertise and scoring may be diminished. Thus, examining the relationship between a direct measure of reviewer expertise and the scoring of research applications under unblinded, real-world conditions is an important area not yet explored in the literature, and may have implications on the subjective differences between reviewers.

In an effort to address this gap, we have conducted a retrospective analysis of historical data, utilizing unblinded individual reviewer evaluations of biomedical research applications, as well as their self-reported expertise ratings, for an anonymized funding program. We were then able to measure the effects of subject matter expertise on scoring in a scenario that may be governed by both social networking/status effects as well as bounded rationality. Although the assignments are not randomized and all the reviewers had some level of relevant expertise, there is sufficient variation in expertise scoring to examine this relationship in a real-world setting. In addition, this analysis includes data from both funded and unfunded applications, examining a broad range of application quality, albeit through a subjective measure. In this work, we did not directly measure the social network links between reviewer and applicant. However, we did examine reviewer and applicant demographic factors, as they may affect the relationship between expertise and scoring. Based on the importance of reviewer expertise to the peer review process, we hypothesize that self-assessed reviewer expertise ratings will closely approximate keyword based measures of intellectual distance and despite exposure to social effects, research close in subject matter will elicit more negative evaluations from reviewers, although there may be potential influences from demographic factors.

## Background

### American Institute of Biological Sciences

The American Institute of Biological Sciences (AIBS) is a national scientific organization that promotes the use of science to inform decision-making that advances biology for the benefit of science and society. For over 50 years, AIBS has provided independent peer review services for funding organizations and research institutes and has worked to identify and promote best practices in peer review through the analysis of peer reviews we have conducted. The data contained in this analysis was generated through an independent peer review AIBS conducted for an unnamed research-funding program.

### Research Funding Program

Research applications were submitted throughout the year to a general program announcement for an anonymized biomedical research-funding program and were reviewed individually as they were received. No formal budget limitations were included in this announcement, although the appropriateness of the proposed budget was a review criterion. Project timelines were limited to 5 years. Topic areas varied considerably across the field of biomedicine, including but not limited to infectious diseases, traumatic injury, physiological and psychological health, rehabilitative medicine, medical simulation, health informatics, medical robotics, and nanomedicine. AIBS coordinated independent, objective peer review of these applications for the research funder. The submitted applications typically had project narrative page lengths of 15–20 pages (the inclusion of biosketches, appendices, etc, often brought total page lengths to 50–75 pages) and had a median budget of $1.4 million. Some were multi-institutional applications. No formal payline (e.g. scoring threshold) was established by the funding agency and decisions were made not only on the basis of scientific merit but also other programmatic factors (e.g. portfolio balance, etc.). AIBS did not take part in funding decisions, nor did we have access to progress or productivity reports from funded applications.

### AIBS Peer Review Process

Similar to a journal-style review process, two reviewers were recruited by AIBS to provide an independent assessment of each application. AIBS staff assessed the research areas covered in the application and invited potential reviewers with appropriate and relevant scientific expertise. Based on AIBS experience, reviewers accepted invitations based largely on how well their expertise matched the application, whether there was a conflict of interest and their availability to participate. Potential reviewers received the application title, abstract and name of the principal investigator to aid in their decision. For this program, reviewers were typically only recruited for one application at a time; if they agreed to review they received a very small honoraria for their participation. If a reviewer accepted the invitation to review, they submitted to AIBS an up-to-date version of their CV and signed a confidentiality agreement. In addition, they declared any conflict of interest and signed a conflict of interest form. AIBS vetted the reviewers for any additional potential conflicts with respect to the application submitted. Once vetted, reviewers were sent the application and a form and guide (review template) for evaluating the application (two reviewers in total for each application). Reviewers typically returned their evaluations within a couple of weeks. The opportunity to discuss the application between reviewers was not given in these reviews. This analysis includes 1,450 reviews of 725 applications (619 applicants) by 1,044 reviewers conducted from 2009 to 2012.

Over this time period, the review process was consistent, using essentially the same review criteria: appropriateness of the research goals and hypotheses, feasibility and appropriateness of the methods and experimental design, the qualifications of the personnel, human subject and animal welfare concerns, the suitability of the facilities, the appropriateness of the budget, and the potential impact of the proposed research. Reviewers utilized these criteria to give the application an overall scientific merit (SM) score on a scale of 1.0 to 5.0 (where 1 is the highest merit and 5 is the lowest merit) as well as rated their own reviewer expertise (RE) relative to the application they reviewed on a scale of 1.0 to 5.0 (where 1 indicates the highest level of expertise and 5 indicates the lowest level of expertise). Reviewers also provided written evaluations following a form and guide template based on the review criteria. Evaluative comments were provided under each review criterion. Guidelines for scoring scientific merit and reviewer expertise are listed in Table 1.

**Table 1:** Definitions for Scientific Merit and Reviewer Expertise Scoring.

**Definitions for Scientific Merit Scoring**	
**Scientific Merit Score**	**Description**
1.0–1.9	EXCEPTIONAL: The scientific merit of the proposal probably places it in the top 10% of proposals in its area of research; it warrants the highest priority for support. This category should be used only for truly outstanding proposals. A score of 1 indicates a very high level of scientific merit.
2.0–2.9	GOOD: The scientific merit of the proposal is such that it warrants high priority for support. A score of 2 indicates a significant level of scientific merit.
3.0–3.9	FAIR: The scientific merit of the proposal is not impressive, and it is probable that it does not warrant support as submitted. If the topic of the proposal is of particular interest, partial support may be warranted. Full support is unlikely to be appropriate. A score of 3 indicates only a moderate level of scientific merit.
4.0–4.9	DEFICIENT: The scientific merit of the proposal is low. The proposal is flawed, and support is unlikely to be justifiable. A score of 4 indicates a low level of scientific merit.
5.0	REJECT: The proposal has very serious deficiencies; it should not be supported under any circumstances. A score of 5 indicates a rejection of the work by the reviewers.
**Definitions for Reviewer Expertise Scoring**	
**Reviewer Expertise Score**	**Description**
1.0–1.9	The proposal is in your specific area of active research. Your knowledge of current publications is thorough.
2.0–2.9	The proposal is in your general area of active research. Your knowledge of the literature is reasonably current. You could apply the techniques of the proposal with little difficulty. You have some ongoing communication with workers in the area of the proposal.
3.0–3.9	The proposal is outside your general area of active research, but it is related. You have knowledge derived from interest in the major discipline embracing the specific proposal, but have little or no contact with other workers active in similar research.
4.0–4.9	The proposal is not related to your active interest and is no more than peripheral to your major discipline.
5.0	The proposal is not related to your major discipline, and your knowledge is only derived through supplemental reading and interest in general science.

## Approach

### Data Gathering and Reduction

To explore the relationship between SM and RE and how this was affected by both reviewer and investigator demographics, such as gender, seniority level, etc., these characteristics needed to be gleaned by hand by one person combing through applicant and reviewer CVs submitted at the time of review. Attributes were then cross-checked by a second person to reduce any potential errors. The reviewer and PI position levels were assessed largely through their job title, (e.g. assistant professor title was labeled as a junior position, while both associate and full professor titles were labeled as non-junior positions). Titles that did not fall into these categories were subject to further review by both persons. If a clear consensus could not be achieved the data were excluded. In a similar process, the reviewer and PI’s sector (academic or non-academic) was largely assessed through the institution title. Again, if institutional titles did not clearly fall into categories, they were subject to further review by both AIBS staff, and if a clear consensus could not be achieved, the data were excluded. Overall, 18 applications and their associated critiques were removed from the data set due to the lack of complete data.

### Non-Random Selection of Reviewers and Application Quality

AIBS recruited reviewers for this program based on relevant expertise, so that the most qualified reviewers evaluated each application. As mentioned above, this analysis represents a retrospective examination of data taken as part of a contract with a funding agency to conduct independent peer review of research applications. Therefore random assignment of reviewer expertise to applications, and therefore control groups with non-expert reviewers, were not possible. As 90% of reviewers have reviewer expertise levels of 2.0 or better in this analysis, it is clear reviewers self-select for participation based on having relevant expertise (which they base on the abstract text, the PI’s name and the fact that they have been selected by AIBS). However, we feel the likelihood of reviewers self-selecting based on application quality is highly unlikely, as the only information they have prior to agreeing to review is the abstract, which likely does not contain enough information for reviewers to assess quality. Also, we find it unlikely that AIBS staff could select for application quality in the assignment process, as assignments are guided largely through expertise matching and conflict of interest vetting. In addition, reviewer recruitment based on expertise represents the standard in peer review practice, and randomization of assignments may in fact introduce decision-making processes not typically present in the expert evaluation of research applications. Nevertheless, it should be mentioned that a potential limitation of this work is the lack of an ex-poste measure of proposal quality, which we are missing as we do not have access to the final productivity reports of funded applications. Further, it is very difficult to measure the ex-poste quality for unfunded applications.

### Variables

**Fig 1:**
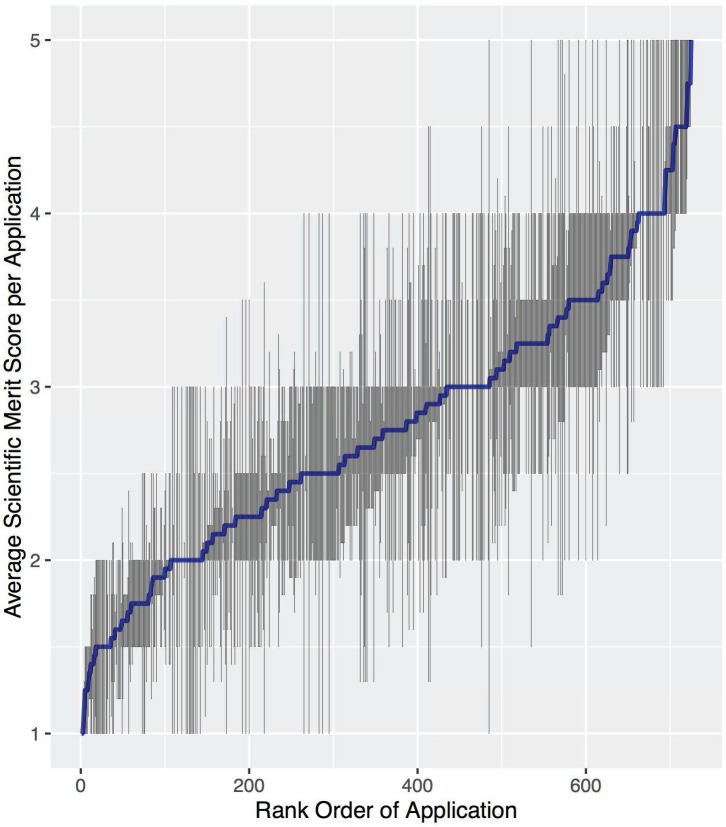
Average SM Score per Application. Average SM score for each application versus the rank order by average SM score, with error bars representing standard error (2009–2012).

Scientific merit was the main dependent variable, with a global mean of 2.77 and a standard deviation of 0.98. A plot of the average SM (plus or minus the standard error) against the relative rank of each application is displayed in Fig 1, underscoring the great variability in evaluations. The main relationship we investigated was between scientific merit and the self-assessed RE, which had a global mean of 1.66 and a standard deviation of 0.54. Other independent variables were categorical in nature, included both reviewer and investigator characteristics and were coded as 0 or 1. These included the position level (1 = presence of junior level; RevJ or PIJ), gender (1 = presence of female; RevF or PIF), academic sector (1 = presence of non-academic sector; RevNonAc or PINonAc), and degree (1 = presence of MD degree, RevMD or PIMD). The overall demographics and relative proportions for reviewers and applicants are listed in Table 2.

**Table 2:** Reviewer and applicant demographics (2009–2012).

	Reviewer Demographics (Total Reviewers = 1044)		Applicant Demographics (Total Proposals = 725)	
Factors	N	%	N	%
Male	799	77	619	85
Female	245	23	106	15
Junior Position	276	26	121	17
Non-Junior Position	768	74	604	83
Academia	965	92	470	65
Non-Academia	79	8	255	35
No MD Degree	716	69	474	65
MD Degree	328	31	251	35

### Multilevel Model and Reliability

Due to the hierarchical structure of the data, a multi-level multiple regression approach was applied, whereby individual reviewer evaluations (level 1) were nested in application groupings (level 2). A random intercept model was used whereby Y_ij_ represents the scientific merit of reviewer *i* of the *j*th application, X_hij_ represents a vector of *h* independent variables with *h* coefficients (β_h_), β_0_ represents the constant intercept component; μ_0j_ represents the random intercept component which varies across applications and the residual error component is represented by ε_ij_. Thus the model is written as:

$$Y_{ij}=\;X_{hij}\beta_{h}+\;\beta_{0}+\;\mu_{0j}+\;\epsilon_{ij}$$ (1)

with ε_ij_ ~ N(0, σ_ε_^2^) and u_0j_ ~ N(0, σ_u_^2^). The variances of the model are var(μ_0j_) = (σ_μ_)^2^ for between application variance and var(ε_ij_) = (σ_ε_)^2^ for residual variance. Using these, we can calculate ρ, the intraproposal correlation coefficient [29], using the following formula:

$$\rho =\frac{\sigma_{\mu }^{2}}{\sigma_{\mu }^{2}\;+\;\sigma_{\epsilon }^{2}}$$ (2)

This represents the correlation between two ratings of the same application. The inter-rater reliability (IRR), which is the reliability of the average rating of an application, can then be calculated using ρ and the Spearman-Brown formula [30].

#### Analytic approach

For comparison, we started with a random intercept-only model as a baseline (model 1):

$$Y_{ij}=\;\beta_{0}+\;\mu_{0j}+\;\varepsilon_{ij}$$ (3)

We successively added the variables to the model (RE [model 2], reviewer demographic variables as a set [model 3], and applicant demographic variables as a set [model 4]) and used the deviance (as measured by -2 log likelihood) as a measure of significant improvement in fit. In this way, internal comparisons can be made without formal controls for expertise. It should be noted that the deviance reported for model 1 was measured against a fixed intercept model with no random component. Similarly, calculation of the R^2^ was based on log likelihood comparisons to the fixed intercept, non-random model [31]. For models 3 and 4, both main effects and interactions with RE were included for every variable. All multilevel models in this paper were based on the maximum likelihood estimation of the linear mixed effect function in R [32]. Reviewer expertise scores were centered by creating Z-scores based on the global RE mean and standard deviation. To visualize the effects of individual independent variables on the SM/RE relationship, scatterplots with simple regression were created. Also, to examine SM variance over the RE range, data were binned into five groupings of RE scores (1.0–1.4 [N = 500], 1.5–1.9[N = 233], 2.0–2.4[N = 604], 2.5–2.9[N = 71], 3.0–3.5[N = 39]); SM variances were calculated for each and values were then plotted against RE bin values.

### Inter-reviewer Agreement

A key component to the bounded rationality hypothesis is the tendency of reviewers to detect weaknesses over strengths. As previous research has suggested there is more agreement on unfundable applications than fundable ones [9,10], we explored the intersection of expertise, proposal quality and inter-reviewer agreement. We examined the difference in score (absolute values) between the two reviewers averaged across applications for reviewer pairs with both high and low average expertise (high is defined as the average RE of the two reviewers being less than or equal to the global median of 1.65; low is defined as average RE that is more than the median). Utilizing an arbitrary funding threshold of an average SM score of better than 2.0 (top 15% of applications) to generate a distinction of application quality, we could then examine these differences for “fundable” (high quality) versus “unfundable” (low quality) applications. In addition, we also examined the proportion of applications where both reviewers agreed on the fundability status for both high and low quality applications and high and low levels of reviewer expertise.

## Results

### Partition of Variance and Reliability

The results of the baseline model fit (model 1) suggest a substantial amount of variation in SM scores across applications, (σ_μ_)^2^ = 0.219±0.042, as well as a substantial residual component (σ_ε_)^2^ = 0.740±0.023 (Table 3). Thus, 22.9% of total variance in the SM score is due to the applications while the majority of variance, 77.1%, is due to a combination of the reviewers, the interactions of reviewers and applications and random noise. This relative proportion of variance is similar to that others have seen and is consistent with the variability we see in Fig 1 [29]. In addition, the intraproposal correlation coefficient (ρ) and the IRR can be calculated from the baseline variances, yielding 0.23 and 0.37, respectively. Both values indicate poor reliability.

**Table 3:** Multi-level regression comparison of random-intercept models.

	Model 1	Model 2	Model 3	Model 4	Model 5
	Baseline Across Applications	RE	RE + Reviewer Demographics	RE + Reviewer and Applicant Demographics	RE + Seniority + Sector
**Random Effects**					
Variance Across Proposals	0.219 (0.042)*	0.213 (0.042)*	0.204 (0.042)*	0.194 (0.042)*	0.192 (0.043)*
Residual Variance	0.740 (0.023)*	0.722 (0.023)*	0.717 (0.023)*	0.714 (0.023)*	0.716 (0.023)*
**Fixed Effects**					
Intercept	2.77 (0.03)*	2.77 (0.03)*	2.80 (0.04)*	2.74 (0.05)*	2.73 (0.04)*
Reviewer Expertise		-0.15 (0.02)*	-0.09 (0.04)*	-0.09 (0.05)	-0.09 (0.03)*
**Deviance (previous model)**					
Change in 2LL	38.9*	36.8*	20.9*	16.0*	36.9* (compared to Model 2)
**R**^**2**^	0.026	0.051	0.070	0.075	0.075

Analysis based on z-score of RE. Asterisk indicates statistical significance (p<0.05). Standard error is reported in parentheses. Each model was compared to the previous model (unless noted otherwise) through the calculation of deviance, as measured by the change in -2 log likelihood. All main effects and interactions with RE were included. Model 1 was compared to a fixed intercept model.

### Reviewer Expertise

**Fig 2:**
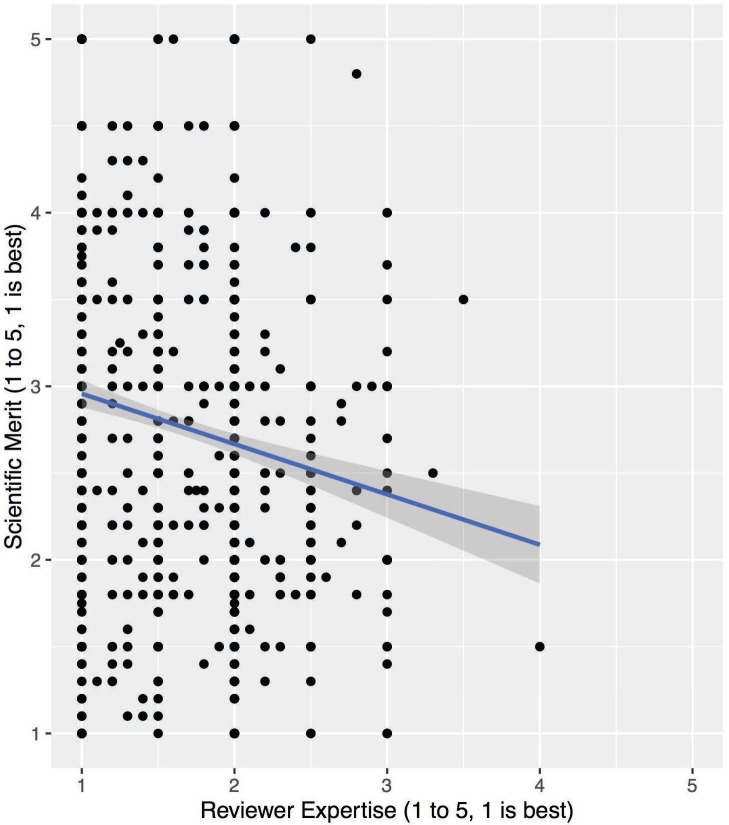
Scatterplot of SM versus RE. Scatterplot and linear regression fit of SM versus RE data with gray area representing 95% confidence intervals (2009–2012).

The addition of the RE variable in model 2 represented a highly significant improvement over the baseline fit (Table 3; χ^2^ (1) = 36.8, p<0.001). There is a significant decrease in variance across proposals (2.7%) and residual variance (2.4%) when reviewer expertise is controlled for, suggesting it is an important source of variation. We also see that the estimate for the centered RE coefficient (z-score of RE) is statistically significant and is negative (-0.15±0.02; p<0.001), whereby lower levels of expertise (higher RE values) result in improving SM scores (lower SM values). Thus, across the entire range of differences in RE in this data set, there is a resulting difference in SM score of 0.84, underscoring the importance of this factor. As visualized in the fitted scatterplot in Fig 2, there is a clear linear, negative relationship between SM and RE, with no obvious step-functions or other non-linearities. The residuals from the regression have no correlation with RE (R^2^<0.001) and are centered around zero (data not shown). Overall, these data are consistent with the data of Boudreau et al. (2016) [17].

However there is a great amount of variability. If the SM data are binned using the procedure described above, a simple regression yields a correlation between SM variance and RE scores (slope = -0.28±0.08, intercept = 1.37±0.17, R^2^ = 0.80, p = 0.04; S1 Fig), while no such correlation was found by Boudreau et al., who suggests greater scoring variance for close research may be a sign of strategic motivations in reviewer scoring [17]. However, this trend is consistent with our reliability analysis that (despite higher agreement on fundability) there are larger differences in score for poorly rated proposals as compared to well-rated ones (see below). Thus, as higher expertise tends to yield poorer scores, there is also more variability.

### Reviewer and Applicant Demographic Factors

**Fig 3:**
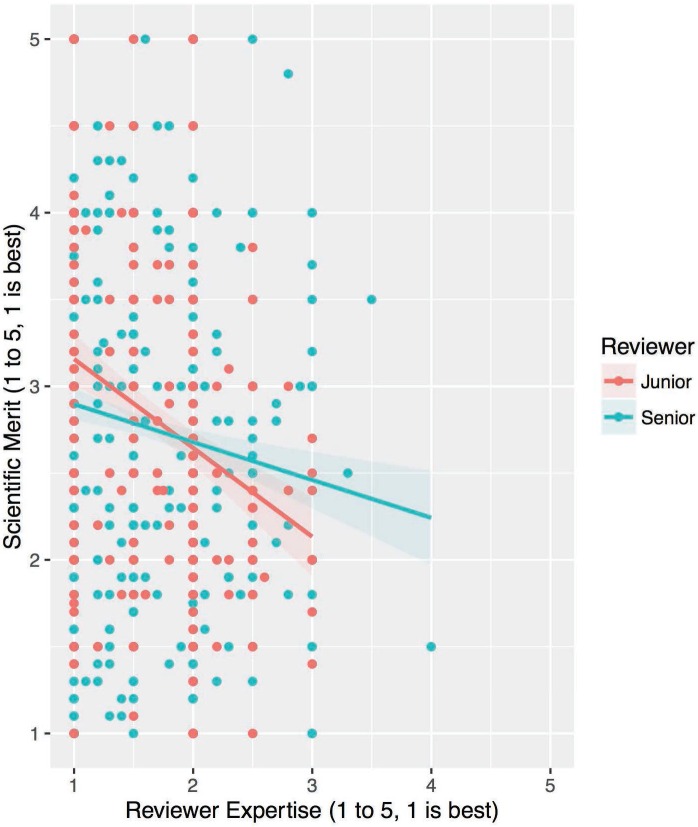
Reviewer Seniority Scatterplots of SM versus RE. Scatterplot and linear fit of raw SM versus RE scoring data of all evaluations by junior reviewers (in red) and by senior reviewers (in blue). The shaded area represents 95% confidence interval.

Reviewer demographic factors (including RevJ, RevF, RevNonAc and RevMD) and their interactions with RE were added in model 3, which again represented a significant improvement in fit over model 2 (Table 3; χ^2^ (8) = 20.9, p = 0.007). The variance across applications and the residual variance decreased, explaining 4.1% and 0.7%, respectively. Research sector (RevNonAc) was found to yield a direct effect (-0.30±0.09, p = 0.001) as did the interaction between reviewer seniority (RevJ) and RE (-0.15±0.06, p = 0.011). When the data are separated by seniority group, plotted and then fit via simple linear regression (Fig 3), we can begin to visualize the effect of reviewer seniority on the relationship between RE and SM scoring, with senior reviewer scoring less sensitive to expertise. These results suggest that reviewer attributes can be important in modifying the relationship between RE and SM. This is in contrast to the results of Boudreau et al. (2016), who found no influence of reviewer seniority on the relationship between intellectual distance and scoring [17].

**Fig 4:**
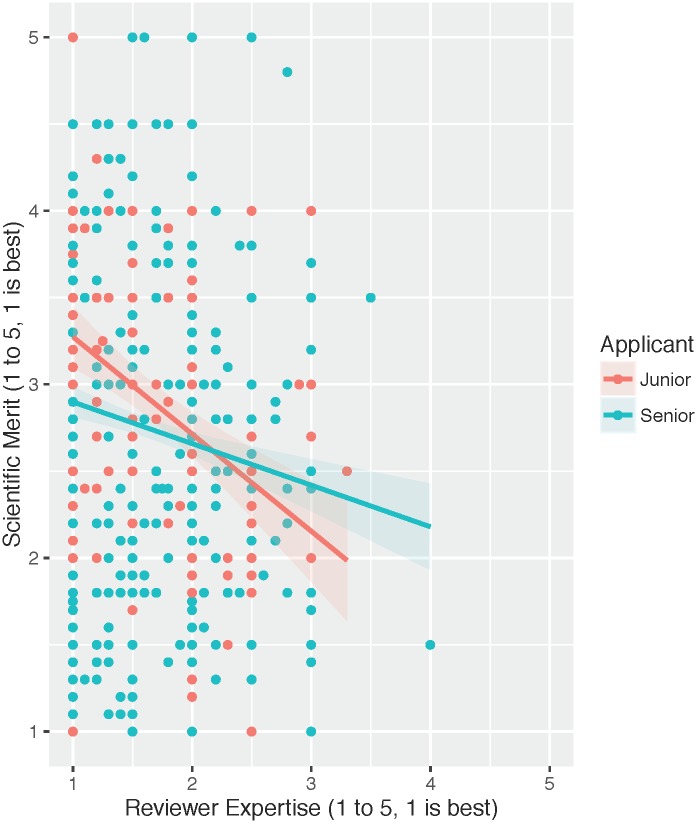
Applicant Seniority Scatterplots of SM versus RE. Scatterplot and linear fit of raw SM versus RE scoring data of all evaluations of junior applicants (red) and non-junior applicants (blue). The shaded area represents 95% confidence interval.

When applicant demographic factors (including PIJ, PIF, PINonAc, and PIMD) and their interactions with RE were added in model 4 (Table 3), a significant improvement over model 3 was observed (χ^2^ (8) = 16.0, p = 0.043). The variance across applications and the residual variance decreased in this case as well, explaining 4.6% and 0.4%, respectively. Both the main effect of PIJ (0.17±0.08, p = 0.02) and its interaction with RE (-0.15±0.07, p = 0.03) were found to be significant. The interaction of RevJ with RE was also still found to be significant in this model (-0.14±0.06, p = 0.02), as was the main effect of RevNonAc (-0.29±0.09, p = 0.001). Triple interactions between RevJ, PIJ and RE as well as interactions directly between PIJ and RevJ were not significant if added to model 4 (χ^2^ (2) = 2.50, p = 0.29). Nevertheless, these data also suggest that seniority of applicants as well as reviewers can influence the relationship between SM scoring and RE. This is visualized in Fig 4, where scoring for senior applicants is less dependent on reviewer expertise. Also, as reviewer research sector (RevNonAc) is still significant in this model, we added an interaction with applicant research sector (PINonAc) to model 4, which yielded an improvement in the model (χ^2^ (1) = 5.19, p = 0.023) and a significant interaction (0.40±0.18, p = 0.024).

In our final model (model 5), we added the significant demographic factors (PIJ and RevJ and their interactions with RE as well as RevNonAc and PINonAc and their interaction) to the basic model 2 (Table 4). There is a clear improvement in fit over model 2 (χ^2^ (7) = 36.9, p<0.001). Significant main effects were seen for RE (-0.09±0.03; p = 0.01), PIJ (0.16±0.07; p = 0.03), RevNonAc (-0.48±0.12; p = 0.0001) and significant interaction effects were seen for RE:RevJ and RE:PIJ (-0.15±0.06; p = 0.01 and -0.16±0.07; p = 0.02, respectively) as well as for RevNonAc:PINonAc (0.41±0.18, p = 0.02). These data suggest that reviewer and applicant seniority as well as research sector can affect SM scoring.

**Table 4:** Summary of Model 5 (RE + Seniority + Research Sector).

**Random Effects**	
Variance Across Proposals	0.192 (0.043)*
Residual Variance	0.716 (0.023)*
Intra-proposal Correlation	0.21*
Inter-Rater Reliability	0.35
R^2^	0.075
**Fixed Effects**	
*Main*	
Intercept	2.73 (0.04)*
Reviewer Expertise (RE)	-0.09 (0.03)*
Junior Reviewer (RevJ)	0.05 (0.06)
Junior Applicant (PIJ)	0.16 (0.07)*
Non Academic Reviewer (RevNonAc)	-0.48 (0.12)*
Non-Academic Applicant (PINonAc)	0.07 (0.06)
*Interactions*	
Reviewer Expertise * Junior Reviewer	-0.15 (0.06)*
Reviewer Expertise * Junior Applicant	-0.16 (0.07)*
Non Academic Reviewer (RevNonAc) * Non-Academic Applicant (PINonAc)	0.41 (0.18)*

Model 5 (random intercept model including fixed effects from RE, RevJ, PIJ, PINonAc and RevNonAc) coefficient estimates are listed. Analysis is based on z-score of RE. Standard error is reported in parentheses and asterisks indicate statistical significance (p<0.05). Results are broken out by random and fixed components (including both main and RE interaction effects). In addition, estimates of intra-proposal correlation and inter-rater reliability are provided.

### Inter-Reviewer Agreement

We also examined the level of agreement between reviewers on the fundability of individual applications based on a hypothetical scoring threshold of SM < 2.0. As seen in Table 5, reviewer pairs of both higher and lower average expertise agree much more on the fundability status of poorer applications (81–82% agreement) as compared to the top scoring applications (33–35% agreement). This is generally consistent with the previous literature that reviewers focus and agree more readily on weaknesses, although somewhat surprising that expertise has no effect [9,10]. To explore this further, we examined the average scoring differences (absolute differences) of high and low expertise reviewer pairs for fundable and unfundable applications. Here we see the exact opposite effect whereby bigger scoring differences (less agreement) are seen amongst poorer applications. However, the scoring range covered by unfundable applications (2.0–5.0) is larger than that for fundable (1.0–1.9), and while reviewers are not aware of a specific scoring threshold for funding, there is less disagreement about fundability between a Fair (3.0) and Deficient (4.0) rating, then there is between an Excellent (1.0) and a Good rating (2.0). It also should be noted that there is slightly worse agreement (via average score differences) for reviewer pairs with higher expertise as compared to low expertise, suggesting that scoring translation is exacerbated in expert reviewers. Thus, while both high and low expertise reviewer pairs agree more on what is unfundable, there is still great variability in the scoring, even among reviewers with high expertise.

**Table 5:** Reviewer scoring and fundability agreement.

	Agreement on Fundability (Higher Expertise)	Agreement on Fundability (Lower Expertise)	Average Score Difference (Higher Expertise)	Average Score Difference (Lower Expertise)
**Fundable Application (Top 15%)**	33%	35%	0.66 (0.05)	0.57 (0.05)
**Unfundable Application (Bottom 85%)**	82%	81%	1.09 (0.05)	0.95 (0.04)

Inter-reviewer agreement between two reviewers assigned the same application on fundability, based on a 2.0 funding threshold (less than 2.0 is arbitrarily deemed fundable). This is shown for fundable and unfundable applications and for higher and lower average reviewer expertise (high is higher than median RE of 1.65; low is lower than median). Also average scoring difference (absolute differences) between assigned reviewers is shown with a similar breakdown (standard error shown in parentheses).

## Discussion

Our results indicate a low IRR and a low proportion of total variance in SM scores due to the applications themselves. This result and other similar findings in the literature underscore the importance of identifying significant predictor variables to help explain this variance and the underlying differences in individual reviewer decision-making [3,8,9,29,33].

In this multi-level analysis, where controlling for self-assessed RE explained 2.7% of variance across proposals and 2.4% of residual variance, we observed RE to be a significant predictor of SM score (Table 3), with a negative and linear correlation (Fig 2). It should be noted that this relationship was observed despite our non-randomized sample, which likely had a smaller range of expertise levels than represented in the Boudreau study, as non-experts were not included. Overall, these results are consistent with the results of Boudreau et al (2016), despite the linear relationship we observed between SM variance and RE, which we feel is explained by the inter-subjectivity of translating similar evaluations into scores across the scoring scale (S1 Fig) [17,34]. Both the regression and the inter-reviewer agreement analysis are consistent with the bounded rationality perspective and the notion that reviewers disproportionally focus on weaknesses, which are detected with greater frequency with increasing levels of subject matter knowledge, thereby creating differences in scoring leniency between reviewers with high or low expertise. As with Boudreau, we can likely discount alternate explanations of competition driving the penalization of close research due to the observed linear relationship between SM and RE, as well as the low agreement by reviewer pairs with high RE on what should be actually be funded.

Despite the unblinded nature of this review, intellectually close applications are still reviewed less favorably than distant ones, which is the opposite result to Li et al (2015). Thus, it is likely that the processes of bounded rationality dominate over social influences, which is an important finding given the many claims of bias and cronyism in peer review. However, based on the multilevel modeling results we report here, it seems reviewer and applicant characteristics explain significant proportions of variance (Table 3) and can potentially alter the relationship between SM and RE (Table 4), which suggests social effects may play out in more subtle ways.

For instance, the sensitivity of SM to RE was diminished as a function of reviewer seniority (Fig 3). It may be that senior reviewers are sampling distinct types of information or there are differences in the weighting of information, systematically (across expertise levels) placing more weight on one review criterion over another. This prioritization of criteria has been described as a commensuration bias [35]. In our unblinded study, it may be that senior reviewers prioritize an investigator’s track record. Li (2015) has observed in NIH review committees that higher applicant-reviewer relatedness (via citation connections) does improve an applicant’s score, the opposite effect of intellectual distance [22]. As senior scientists have more established publication and social networks, it is more likely a senior reviewer is related (by citation) to a given applicant. Although reliance on relatedness may introduce the potential for cronyism in the review process, Li shows evidence that reviewers use relatedness to gather additional information and make inferences about an application’s quality, and on the whole, utilizing relatedness results in a 30% increase in the correlation between funding decisions and application quality. Thus, expert reviewers may be using prior knowledge about an applicant to temper their evaluation of an application, but the extent depends on reviewer seniority.

While it should be noted that Boudreau et al. (2016) did not find that reviewer seniority affected the relationship between scoring and expertise, that review was blinded to applicant information; therefore, consistent with the above explanation, one would not expect any influence [17]. However, Jayasinghe et al. (2003) also investigated assessor seniority as a predictor of evaluation ratings of applications submitted to the Australian Research Council [29]. Assessor seniority was not found to be a significant component to their model, although RE was not controlled for in their model, and it is unclear what proportion of the assessor pool was senior versus junior. They did, however, observe applicant seniority as an important predictor of application ratings.

We also observed applicant seniority to be a significant predictor of scoring, finding both systematic effects as well as interactions with RE, which diminished the sensitivity of SM scoring to RE (Fig 4). In our observation, as well as in Jayasinghe’s study, senior applicants were found to systematically receive more favorable scores as well (Table 4). This is also consistent with the above notion that based on applicant status and placement in social networks, reviewers may be tempering methodological weaknesses with *a priori* knowledge of the applicant. Surprisingly in our model, reviewer and applicant seniority were not found to jointly interact with RE in any significant way, as one would assume senior reviewers and senior applicants would have the highest likelihood for social overlap. It may be more complicated social relationships are at play.

It should also be noted that reviewers from non-academic backgrounds tended to be more generous evaluators, and that this effect was negated if the applicants were also not from academia (Table 4). It may be that certain social network effects are different in non-academic circles due to differences in cultural norms [36]. For instance, competition effects may be more pronounced in a non-academic environment where intellectual property concerns dominate. This and other cultural differences may be important factors in reviewer decision making processes, and need to be explored further, as the sample of non-academic reviewers was relatively small in this study (N = 127).

As mentioned above, the non-randomization of the reviewer sample and the lack of direct measures of application quality are limitations of this analysis, although the likelihood of a selection bias for application quality is deemed low. Also, there are likely several omitted variables, particularly properties of applications like innovation and inter-disciplinarity, that are not included in our analysis. However, Boudreau has reported no interaction between innovation and intellectual distance [17]. Also, interdisciplinary proposals would likely yield lowered expertise assessments, which based on the observed trend would result in more favorable scoring patterns, which again is in contrast to recent reports [37]. Thus, we feel the omitted variables do not adequately explain the trend observed here. Also, previous literature has suggested that reviewer expertise seems to be central to a reviewer’s evaluation, as expressed by Lamont (2009) [16]. The limitations underscore the need for further exploration of the role of reviewer expertise in the decision making process of reviewers.

Future studies must also include more extensive analysis (including individually scored criteria) in prospective trials of blinded and un-blinded reviews with applicant and reviewer demographic factors and expertise as variables. Additionally, the role of individual RE in review panels and how this affected by team dynamics, collective expertise and discussion should be explored. And more direct measure of social connectivity should be employed to explore relationships with self-assessed expertise as well as scoring.

Nevertheless, despite these limitations, we have observed in real world conditions a clear but complex role of reviewer expertise in the research evaluation process. Our results also suggest that, even for reviewer pairs with high expertise, substantial reviewer disagreement exists about the scoring of applications. Given the large amount of variability we observe across reviewers, it may be that there is simply a great diversity of opinion in what good science is and a fundamental limitation in the ability of a reviewer to forecast which projects are the most likely to be successful and impactful. More research exploring the types of weaknesses and strengths reviewers focus on and how they are weighted and prioritized will be crucial in accounting for inter-reviewer disagreement. Many of these results will have great impact on not only understanding the multi-faceted process of decision-making in peer review, but will have practical implications in guiding how reviewers should be recruited, trained, and moderated by administrative staff to produce the most equitable, reliable, and valid evaluations.

## Supporting Information

S1 Fig SM Variance versus Binned RE. SM scoring data was binned according by RE into 5 groups and then the variance in SM score of these groups was plotted against RE. A linear regression fit of the data is displayed.
(TIFF)

S1 File Anonymized Source Data File. Anonymized scoring and demographic data for the review of each application have been compiled in a CSV file.
(CSV)
