# Supplementary figures and images for: The Influence of Peer Reviewer Expertise on the Evaluation of Research Funding Applications
Source: PLoS One. 2016 Oct 21;11(10):e0165147. doi: 10.1371/journal.pone.0165147 (PMC5074495; doi:10.1371/journal.pone.0165147)

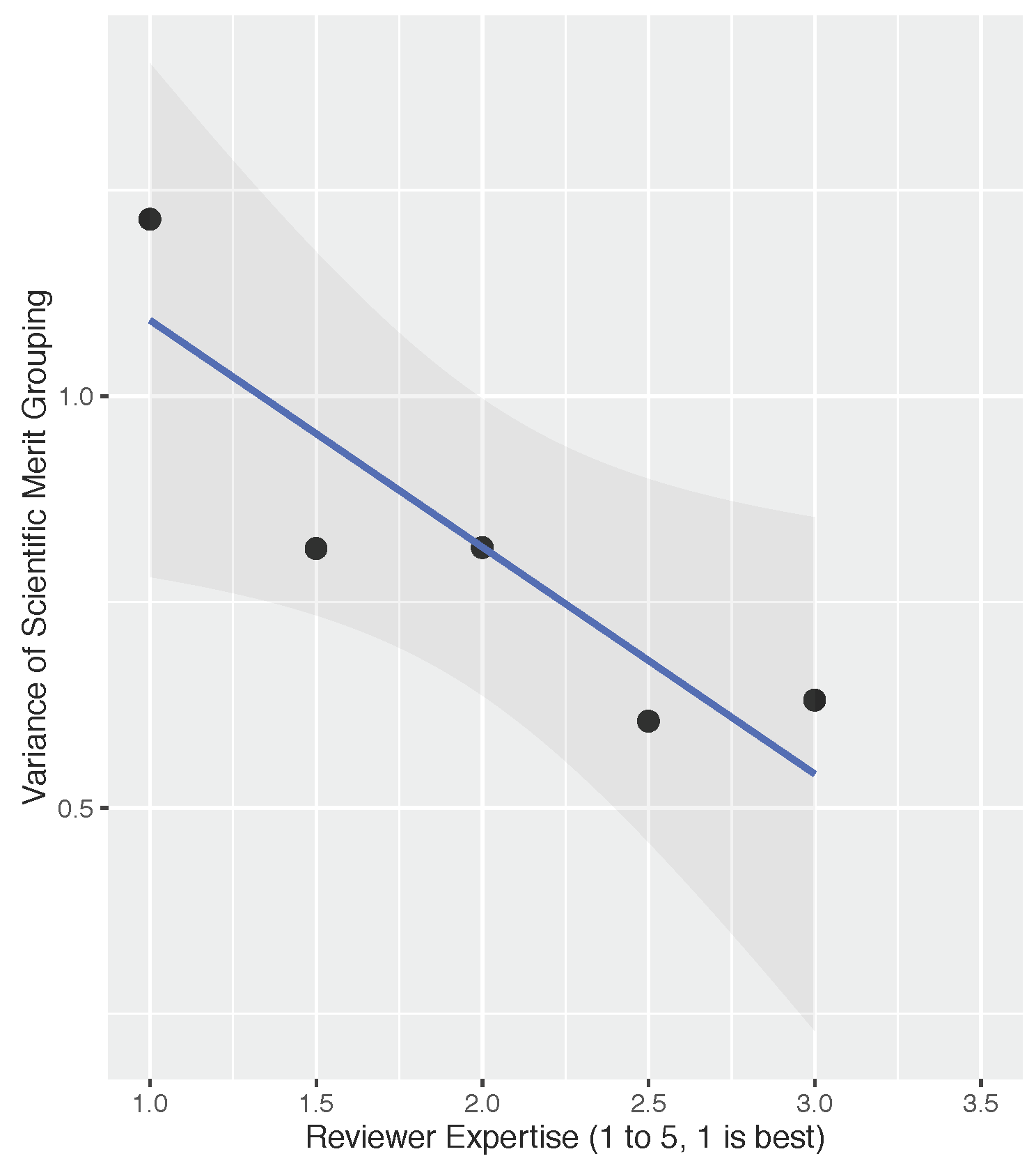

Supplement: S1 Fig — SM scoring data was binned according by RE into 5 groups and then the variance in SM score of these groups was plotted against RE. A linear regression fit of the data is displayed. (TIFF) [file pone.0165147.s001.tiff]
